# Heights and spatial relationships of the facial muscles acting on the nasolabial fold by dissection and three-dimensional microcomputed tomography

**DOI:** 10.1371/journal.pone.0237043

**Published:** 2020-08-04

**Authors:** Mi‐Sun Hur, Jehoon O., Hun‐Mu Yang, Hyun‐Jin Kwon, Seunggyu Lee, Ha Seong Lim, So Young Lim, Chang‐Seok Oh

**Affiliations:** 1 Department of Anatomy, Catholic Kwandong University College of Medicine, Gangneung, Korea; 2 Department of Anatomy, Yonsei University College of Medicine, Seoul, Korea; 3 Department of Mathematics and Research Institute of Natural Science, Gyeongsang National University, Jinju, Korea; 4 Areumdaunnara Dermatology Clinic, Seongnam, Korea; 5 Department of Plastic Surgery, Samsung Medical Center, Sungkyunkwan University School of Medicine, Seoul, Korea; 6 Department of Anatomy and Cell Biology, Sungkyunkwan University School of Medicine, Suwon, Korea; New York Institute of Technology, UNITED STATES

## Abstract

The aim of this study was to clarify the heights and spatial relationships of the facial muscles acting on the nasolabial fold (NLF) by dissection and three-dimensional microcomputed tomography for use in aesthetic treatments. This study used 56 specimens from 34 embalmed adult Korean. A reference line (RF) was set to imitate the NLF after removing the skin, from the superior point of the alar facial crease to the lateral point of the orbicularis oris muscle at the level of the corner of the mouth. The heights and spatial relationships of the facial muscles along the RF could be categorized into five main patterns. The dominant pattern was that the levator labii superioris alaeque nasi muscle (LLSAN), levator labii superioris muscle (LLS), zygomaticus minor muscle (Zmi), and zygomaticus major muscle (Zmj) were on the medial third, medial half, middle third, and lateral third of the RF, respectively. In micro-CT imaging, beneath the skin of the medial half of the NLF, the LLSAN and Zmi fibers inserted into the dermis of the NLF and adjacent to the NLF. Beneath the skin of the middle third of the NLF, the Zmi fibers were found before the muscle inserted into the dermis of the NLF and adjacent to the NLF. Beneath the skin of the lateral third of the NLF, the lateral margin of the orbicularis oris muscle and some Zmj fibers were found at the location of the NLF. The present study utilized dissections and micro-CT to reveal the general pattern and variations of heights and spatial relationships of the facial muscles passing beneath the NLF. These findings will be useful for understanding which muscles affect specific parts of NLFs with various contours, for reducing the NLF in aesthetic treatments, and for reconstructing the NLF in cases of facial paralysis.

## Introduction

The nasolabial fold (NLF), commonly known as “smile line” or “laugh line”, begins at the alar groove and alar facial crease and runs down the lateral aspects of the upper lip [1,2]. The NLF is one of the most noticeable signs of facial aging [3]. Young individuals having deep NLFs tend to consider themselves to look older than their age. Depth and prominence of the NLF may also affect and disturb the facial expression. However, its complete elimination can be detrimental to facial harmony [4]. Thus the NLF has been regarded as one of the most important issues for esthetics.

The NLF is not a simple crease, but a complex three-dimensional curvature resulting from the crease which forms the medial boundary of the malar fat pad due to the cutaneous insertion of the upper lip elevators and zygomaticus major muscle (Zmj) along the sulcus. Therefore, the NFL can be defined as one of natural components of the face rather than a wrinkle which appears as a result of aging [2]. The factors influencing the formation of NFL include the loss of skin thickness over the sulcus, the presence of redundant skin drooping over the sulcus, the excessive fat deposits laterally to the sulcus, ptosis and/or laxity of the malar fat pad, and muscular hyperactivity. In elderly individuals, more than one factor is usually related with the prominent NLF, and the NLF can vary from individual to individual [4]. Thus, the area of NLF is a special anatomical region that requires special attention for comprehensive assessment [5]. Successful treatment of the NLF is absolutely dependent on appropriate assessment of what factors are actually creating and deepening the NLF [6].

NLFs can be divided into the following five types based on their anatomical and histological features: skin, fat-pad, muscular, bone retrusion, and hybrid types. These different types of NLFs require different treatments [5].

There has been controversy about whether contraction of the facial muscles is related to the shape of the NLF. Zufferey [7] described that the muscles of smiling are directly responsible for producing the shape and depth of the fold, whereas Pessa et al. [8] found no significant correlation between the pattern of the facial muscles and the length and contour of the NLF. However, several authors have reported that contractions of the facial muscles are closely related to deepening of the NLF. Kane [9] reported that in young to middle-aged patients (aged 30–59 years), a considerable proportion of the fold is caused by muscle contraction. Zufferey [7] reported that the NLF results from muscular activity on their upper lip, either directly or indirectly from tractors acting via the modiolus. Also, Beer et al. [10] confirmed the muscular theory of the cause of the NLF in a histomorphological study.

Repetitive movement of the facial muscles creates a skin crease and induces a dynamic fold [11]. A wrinkle first forms as a dynamic wrinkle and then eventually becomes fixed as a fine line [2]. Hyperfunctional muscles gradually transform dynamic lines into permanent deep static rhytides and crevices [12]. In addition, Rubin et al. [13] and Zufferey [7] described that the NLF disappears in a paralyzed face, but is retained upon death, implying that the facial muscles are a primary etiology in the formation of the NLF.

The most common treatments for correcting NLFs are injectable fillers. However, injecting a toxin can also improve a deepened NLF that appears when smiling or performing asymmetric movements of the face [2]. In some cases, the single application of a filler to the NFL may produce undesirable results, especially when the main factor producing a prominent NLF is muscular hypercontraction. The characteristics of the skin are adversely affected by muscular hyperactivity due to the loss of biomechanical support from structures such as subcutaneous fullness and fat [4]. Thus, contraction of the facial muscles is one of the important targets for reducing the NLF in aesthetic treatments.

Approaches targeting specific facial muscles have been reported when treating the NLF using BoNT-type A (BoNT-A) and surgery. Seo [14] stated that such a selective approach to NLF treatment can be attempted using BoNT-A when smiling exaggerates the severity of the NLF or asymmetry, and that directly injecting BoNT-A into the Zmj is recommended for Zmj-associated NLFs caused by excessive contraction of the muscle resulting in elongation of the NLF up to the lateral cheek. Kane [9] reported that injecting BoNT-A into the levator labii superioris alaeque nasi muscle (LLSAN) improved the NLF. Pessa [15] reported that LLSAN resection softened the medial NLF, while partial resection of the levator labii superioris muscle (LLS) has occasionally been added to further weaken the middle NLF. Wang and Huang [16] reported surgical softening of the NLFs by severing the cutaneous insertions of the mimetic muscles and by applying liposuction. These observations together indicate that contraction of a particular muscle can affect a specific part of the NLF, thereby forming its various contours, and so a selective approach to the facial muscles can be efficient in treating NLF caused by muscle hypercontraction.

Repetitive muscle contraction produces lines on the skin that are perpendicular to the muscle forces [12]. Thus, contractions of the facial muscles passing beneath the NLF can deepen the NLF as well as the muscle fibers inserting into the dermis of the NLF. There has been a focus on the muscles inserting into the dermis of the NLF and their corresponding heights along the NLF [17,18], and the influence of the midfacial muscular pattern on the shape of the NLF [8,19]. However, the patterns of the spatial relationships of the facial muscles passing beneath the NLF and their corresponding heights along the NLF have been rarely investigated, especially in Asians.

The aim of this study was to clarify the heights and spatial relationships of the facial muscles acting on the NLF by dissection and three-dimensional (3D) microcomputed tomography (micro-CT). These findings will be useful for understanding which muscles affect specific parts of the NLF with various contours, and when designing treatments and surgical procedures for the NLF in facial rejuvenation, treating facial asymmetry, and reconstruction of the NLF in cases of facial paralysis when these conditions are caused by contractions of the facial muscles.

## Materials and methods

### Specimens and dissection

This study used 56 specimens from 34 embalmed adult Korean cadavers (15 males and 19 females; mean age, 76.3 years). Forty-four specimens were dissected to analyze the heights and spatial relationships of the facial muscles acting on the NLF. Facial skin just lateral to the NLF was removed and the skin remaining attached to the NLF was reflected to observe the attachments of muscles to the fold. The remaining skin was then removed to reveal the entire muscles related to the NLF. A reference line (RF) was set to imitate the NLF after removing the skin, from the superior point of the alar facial crease to the lateral point of the orbicularis oris muscle (OOr) at the level of the corner of the mouth. To classify the heights and spatial relationships of the facial muscles along the RF, the RF was divided into medial, middle, and lateral parts. The distance from the corner of the mouth to the lateral margin of the OOr was measured using digital calipers.

### 3D micro-CT visualization

Twelve specimens of the facial muscles attached to the skin of the NLF were obtained for micro-CT imaging. The specimens were acquired from the area below the nasal ala to the area above the corner of the mouth. Harvested specimens were fixed in 10% formalin for 2~3 days and then dehydrated in a graded series of 30%, 50%, and 70% ethanol for 1 day each. After dehydration, they were stained in 1% phosphotungstic acid solution with 70% ethanol for 1 week. Micro-CT was performed using a Skyscan 1173 device (Bruker, Kontich, Belgium) at a voltage of 70 kV and a current of 114 μA. NRecon software (version 1.7.0.4, Bruker) was used for data reconstruction, and 3D volume-rendered images were analyzed using CTVox software (version 2.7, Bruker).

### Mathematical simulation of contractions of the facial muscles passing beneath the NLF

We consider that the motions of the fibers of the facial muscles (LLSAN, LLS, zygomaticus minor muscle [Zmi], and Zmj) are governed by elastic forces. The immersed-boundary method is one of the most popular methods for modeling such phenomena [20]. In the present study, the forces generated by each fiber were derived from the elastic energy functionals, and the motion of the muscle was represented as imaginary fluid flows as follows:

$$\begin{array}{l} E[X(\cdot ,t)]=\frac{\sigma }{2}\int {\left(\left|\frac{\partial X}{\partial s}\right|\right)}^{2}ds,\\ \;F(s,t)=-\frac{℘E[X(\cdot ,t)]}{℘X(s,t)},\\ \;U(s,t)=\frac{X(s,t)}{\partial t}=\int u(x,t)\delta (x-X(s,t))dx,\\ \;\frac{Du}{Dt}=-\nabla p+\frac{1}{Re}\Delta u+\frac{1}{We}f,\\ \;\nabla \cdot u=0. \end{array}$$

where *E* is the elastic energy, **X** is the position of a fiber, **F** is the elastic force derived from the variational derivative of *E*, **U** is the velocity of **X**, **u** is the fluid velocity, *D* is the material derivative, *p* is the pressure, f is the fluid force density derived from F, *Re* = ρU*L*/η is the Reynolds number, *We* = ρ(U*)^2^L*/σ is the Weber number, ρ is the density, *s* is the parameter, U* is the characteristic velocity, L* is the characteristic length, η is the viscosity, and σ is the surface tension coefficient. Since it was impossible to measure the velocity and force magnitudes in the present application, we assumed *Re* = 200 and *We* = 20, which are reasonable moderate values for the conditions in the present study [21].

### Ethical approval

All cadavers had been legally donated to Catholic Kwandong University College of Medicine and the Surgical Anatomy Education Centre of the Yonsei University College of Medicine. The present study was conducted in accordance with the Declaration of Helsinki. None of the transplant donors were from a vulnerable population and all donors or next of kin provided written informed consent that was freely given.

## Results

### Heights and spatial relationships of the muscles acting on the NLF in dissections

**Fig 1:**
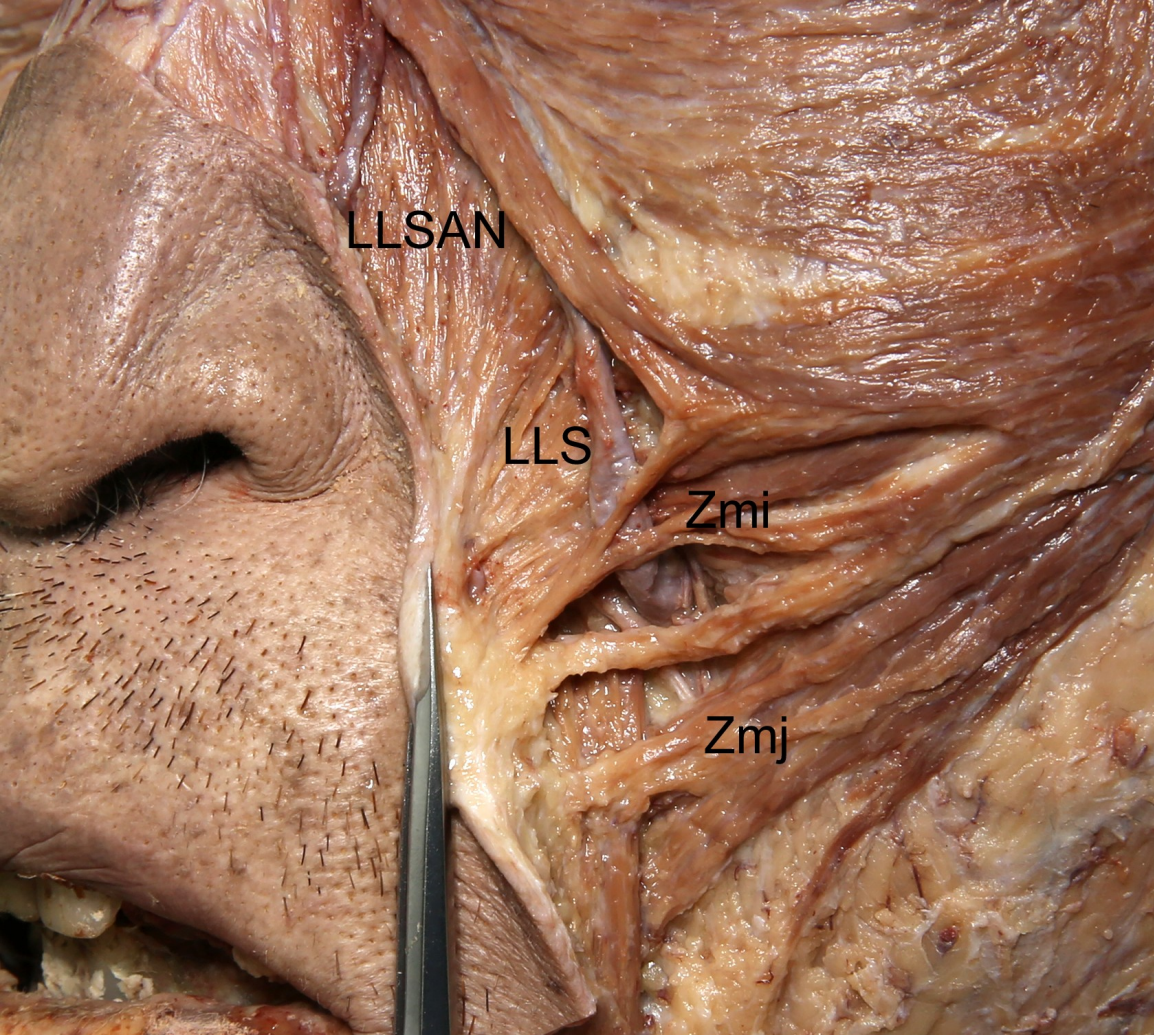
Attachments of the LLSAN, LLS, Zmi, and Zmj to the dermis of the NLF. Some superficial fibers of the LLSAN, LLS, and Zmi were clearly attached to the dermis of the NLF, and a few fibers of the Zmj were attached to the dermis of the NLF. Facial skin just lateral to the NLF was removed and the remaining skin was reflected to reveal the attachments of the muscles to the NLF.

Attachments of the LLSAN, LLS, Zmi, and Zmj to the dermis of the NLF were observed in dissections. Some superficial fibers of the LLSAN, LLS, and Zmi were clearly attached to the dermis of the NLF in all specimens, while a few fibers of the Zmj were attached to the dermis of the NLF in some specimens (Fig 1).

The RF was similar to the lateral margin of the OOr with the muscles (LLSAN, LLS, Zmj, and Zmj) blending with the margin. Thus, the OOr was located in the medial side of the RF, while the LLSAN, LLS, Zmi, and Zmj were located in the lateral side of the RF. The LLSAN was on the medial third of the RF in all 44 dissected specimens (100%). The LLS was on the alare to the midpoint (medial half) and medial third of the RF in 27 specimens (61.4%) and 17 specimens (38.6%), respectively. The Zmi was on the upper part of the middle third of the RF and the middle third of the RF in 22 specimens (50.0%) and 22 specimens (50.0%), respectively. The Zmj was on the lateral third of the RF and the upper part of the lateral half of the RF in 28 specimens (63.6%) and 15 specimens (34.1%), respectively. The Zmj was on the lateral half of the RF in one specimen (2.3%). The Zmj was observed with the levator anguli oris muscle (LAO) along the RF in all specimens.

A bifid Zmj was found in 13 of the 44 specimens (29.5%). In 7 of these 13 specimens, the inferior fibers of the bifid Zmj were slightly narrower than its superior fibers, while the inferior fibers of the bifid Zmj were considerably thinner than its superior fibers in the other 6 specimens. When the bifid Zmj had quite-thin inferior fibers, its main superior fibers were classified at the level of the RF while the quite-thin inferior fibers were not. The inferior fibers of the bifid Zmj appeared toward or below the level of the mouth corner.

**Fig 2:**
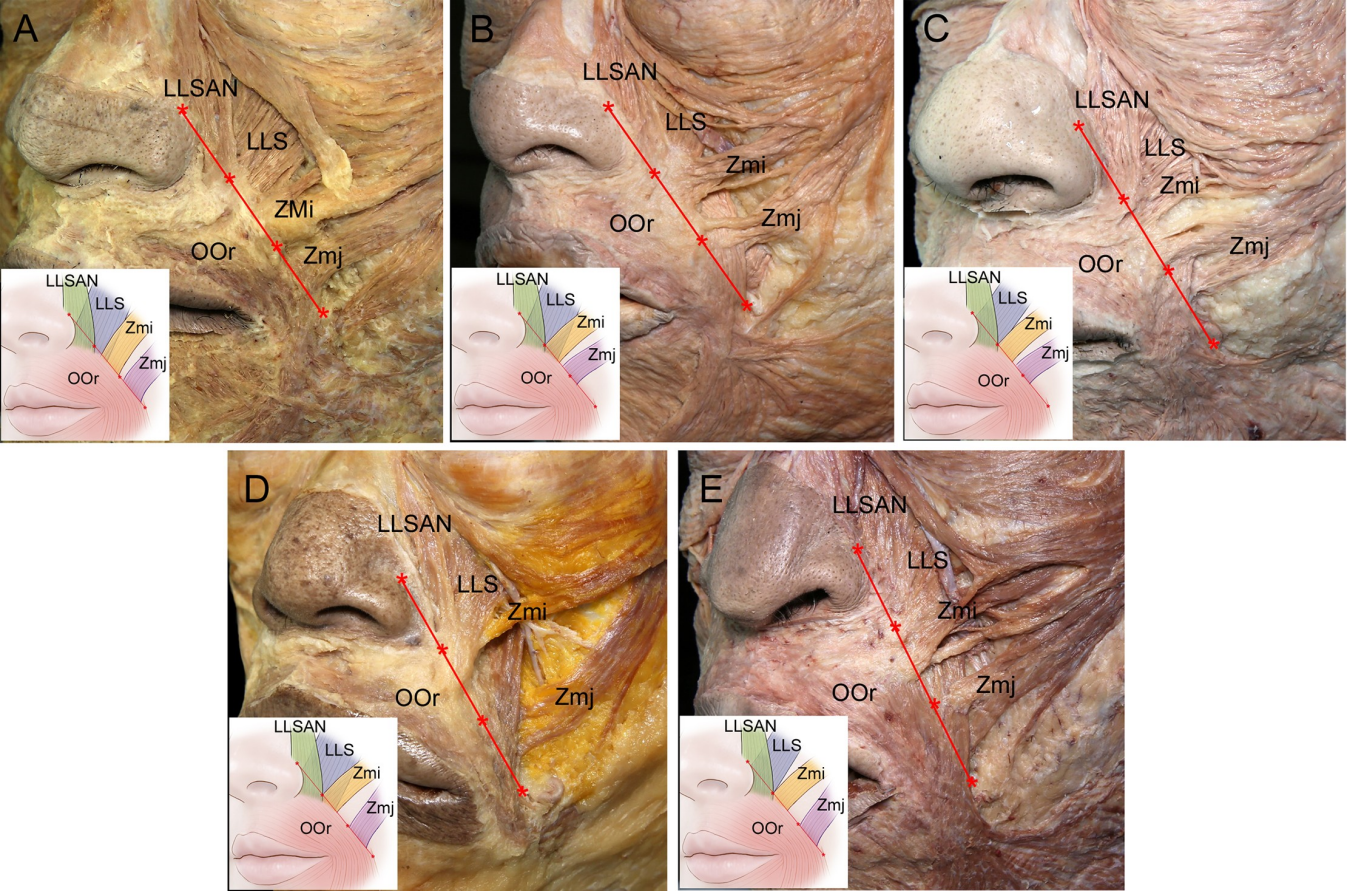
Heights and spatial relationships of the facial muscles along the RF (red) in the five main identified patterns. (A) In type I the LLSAN, LLS, Zmi, and Zmj were on the medial third, medial half, middle third, and lateral third of the RF, respectively. (B) In type II the LLSAN, LLS, Zmi, and Zmj were on the medial third, medial half, upper part of the middle third, and upper part of the lateral half of the RF, respectively. (C) In type III the LLSAN, LLS, Zmi, and Zmj were on the medial third, medial third, upper part of the middle third, and upper part of the lateral half of the RF, respectively. (D) In type IV the LLSAN, LLS, Zmi, and Zmj were on the medial third, medial half, upper part of the middle third, and lateral third of the RF, respectively. (E) In type V the LLSAN, LLS, Zmi, and Zmj were on the medial third, medial third, upper part of the middle third, and lateral third of the RF, respectively. The RF was set to imitate the NLF from the superior point of the alar facial crease to the lateral point of the OOr at the level of the corner of the mouth. The asterisks divide the RF into its medial, middle, and lateral parts.

The heights and spatial relationships of the facial muscles along the RF could be categorized into the following five main patterns (Fig 2):

1. Type I (*n* = 16, 36.4%), in which the LLSAN, LLS, Zmi, and Zmj were on the medial third, medial half, middle third, and lateral third of the RF, respectively.
2. Type II (*n* = 6, 13.6%), in which the LLSAN, LLS, Zmi, and Zmj were on the medial third, medial half, upper part of the middle third, and upper part of the lateral half of the RF, respectively.
3. Type III (*n* = 6, 13.6%), in which the LLSAN, LLS, Zmi, and Zmj were on the medial third, medial third, upper part of the middle third, and upper part of the lateral half of the RF, respectively.
4. Type IV (*n* = 5, 11.4%), in which the LLSAN, LLS, Zmi, and Zmj were on the medial third, medial half, upper part of the middle third, and lateral third of the RF, respectively.
5. Type V (*n* = 4, 9.1%), in which the LLSAN, LLS, Zmi, and Zmj were on the medial third, medial third, upper part of the middle third, and lateral third of the RF, respectively.

Other minor variants were also observed. In three specimens (6.8%) the LLSAN, LLS, Zmi, and Zmj were on the medial third, medial third, middle third, and lateral third of the RF, respectively. In another three specimens (6.8%) the LLSAN, LLS, Zmi, and Zmj were on the medial third, medial third, middle third, and upper part of the lateral half of the RF, respectively. In one specimen (2.3%) the LLSAN, LLS, Zmi, and Zmj were on the medial third, medial third, upper part of the middle third, and lateral half of the RF, respectively. The average distance from the corner of the mouth to the lateral margin of the OOr was 17.4 cm on the left side and 16.8 cm on the right side.

### Arrangement of the facial muscles beneath the NLF in micro-CT imaging

**Fig 3:**
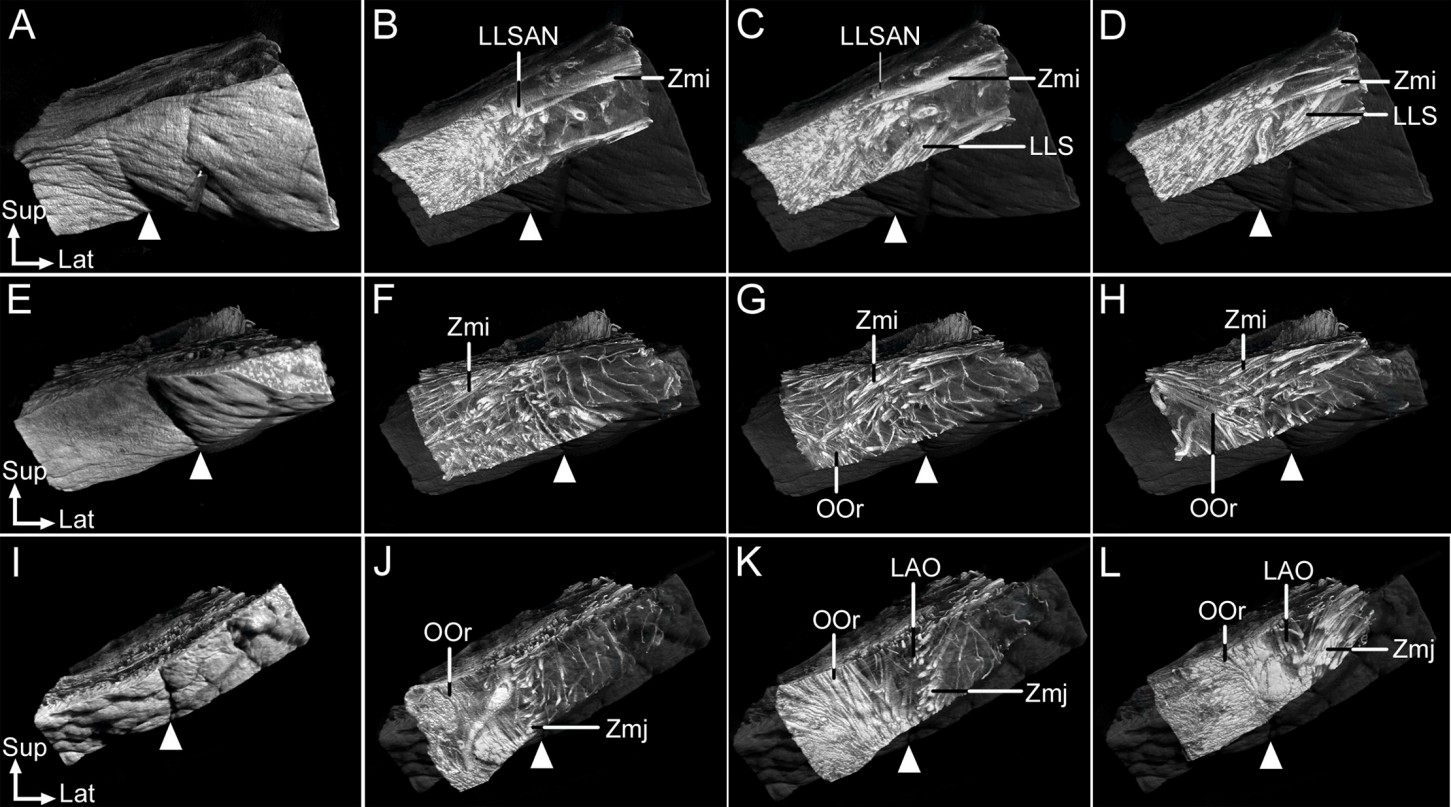
Heights and spatial relationships of the facial muscles acting on the NLF from the skin to the deeper area as revealed by microcomputed tomography. (A–D) The NLF in its medial half (arrowhead) (A). (B) Beneath the skin of the NLF, LLSAN and Zmi fibers inserted into the dermis of the NLF and adjacent to the NLF. (C) At the deeper site of the NLF, the LLSAN, LLS, and Zmi fibers interdigitated together in different directions. (D) At the still-deeper site, more LLS fibers were found with the Zmi fibers. (E–H) The NLF in its middle third (arrowhead) (E). (F) Beneath the skin of the NLF, the Zmi fibers were found before their insertions into the dermis of the NLF and adjacent to the NLF. (G) At the deeper site, more Zmi fibers and some OOr fibers were found. (H) At the still-deeper site, the Zmi fibers intermingled with the lateral margin of the OOr. (I–L) The NLF in its lateral third (arrowhead) (I). (J) Beneath the skin of the NLF, the lateral margin of the OOr and some Zmj fibers were found. (K) At the deeper site, more Zmj and OOr fibers blended with the levator anguli oris muscle. (L) At the still-deeper site, more OOr and Zmj fibers were found with the LAO. At all levels of the NLF, the location of the NLF (arrowheads) corresponded with the lateral margin of the OOr where the LLSAN, LLS, Zmi, and Zmj intermingled and blended with that margin. The skin was made transparent to reveal the deeper areas so that the location of the NLF and the facial muscles beneath it could be compared. Serial images of the facial muscles beneath the NLF are presented in an online video. Lat, lateral; LAO, levator anguli oris muscle; Sup, superior.

Noninvasive serial images obtained by micro-CT confirmed the heights and spatial relationships of the facial muscles along the NLF in detail from the skin surface to deeper areas (Fig 3). Beneath the skin of the medial half of the NLF, the LLSAN and Zmi fibers inserted into the dermis of the NLF and adjacent to the NLF (Fig 3B). At the deeper site of the NLF, the LLSAN, LLS, and Zmi fibers intermingled together in different directions, descending anterolaterally, anteromedially, and lateromedially, respectively (Fig 3C). At the still-deeper site, more LLS fibers were found with the Zmi fibers (Fig 3D) (S1 Video). Beneath the skin of the middle third of the NLF, the Zmi fibers were found before the muscle inserted into the dermis of the NLF and adjacent to the NLF (Fig 3F). At the deeper site, more Zmi fibers and some OOr fibers were found (Fig 3G). At the still-deeper site, the Zmi were intermingled with the lateral margin of the OOr (Fig 3H) (S2 Video). Beneath the skin of the lateral third of the NLF, the lateral margin of the OOr and some Zmj fibers were found at the location of the NLF (Fig 3J). At the deeper site, more Zmj and OOr fibers blended with the LAO (Fig 3K), while more OOr and Zmj fibers were found with the LAO at the still-deeper site (Fig 3L) (S3 Video).

The locations of the NLF corresponded with the locations of the lateral margin of the OOr with the muscles (LLSAN, LLS, Zmj, and Zmj) blending with the margin. In the medial half of the NLF, the LLSAN, LLS, Zmi intermingled and blended with the lateral margin of the OOr in different directions. In the middle third of the NLF, the Zmi intermingled and blended with the lateral margin of the OOr. Finally, in the lateral third of the NLF, the Zmj blended with the OOr and LAO.

## Discussion

**Fig 4:**
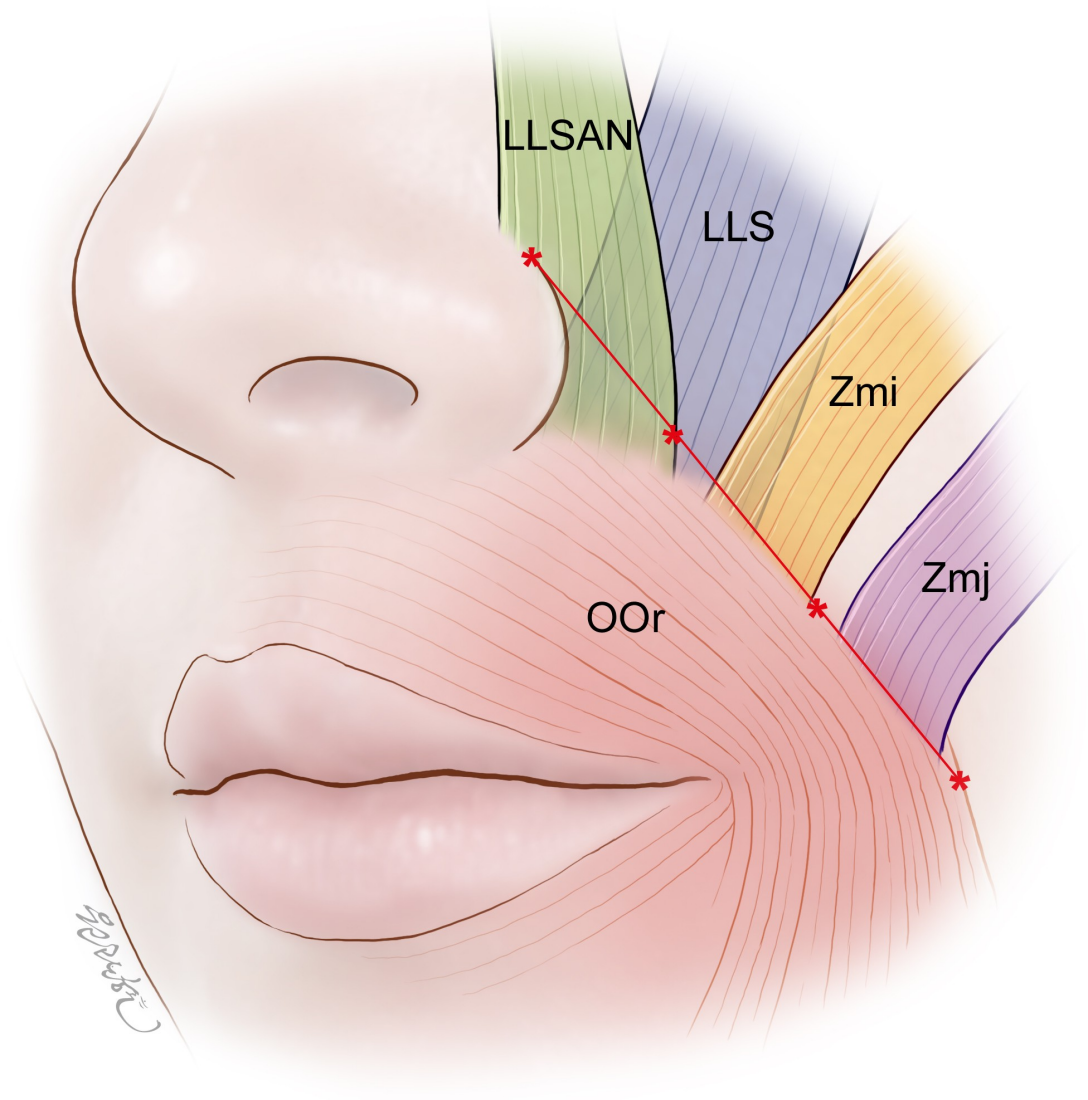
Schematic of the general pattern of heights and spatial relationships of the facial muscles along the RF. **The LLSAN, LLS, Zmi, and Zmj were on the medial third, medial half, middle third, and lateral third of the RF, respectively.** At the location of the RF, the LLSAN, LLS, Zmi, and Zmj intermingled and blended with the lateral margin of the OOr. The RF was set to imitate the NLF from the superior point of the alar facial crease to the lateral point of the OOr at the level of the corner of the mouth. The asterisks divide the RF into its medial, middle, and lateral parts.

This study used dissection and serial micro-CT images to characterize the heights and spatial relationships of the facial muscles along the NLF from the skin surface to deeper areas. These combined methods allowed both internal and external examinations of the NLF in order to obtain more accurate results. Both the RF in dissections and the location of the NLF in micro-CT images corresponded with the lateral margin of the OOr where the LLSAN, LLS, Zmi, and Zmj blended with that margin, implying few anatomical variations between the RF and the location of the NLF. At different heights along the NLF, several muscles intermingled and blended with the lateral margin of the OOr in different directions. These findings can be utilized when treating the NLF with BoNT-A and performing partial resections of selected muscles. The present study has revealed the following dominant pattern of heights and spatial relationships of the facial muscles along the RF (Fig 4):

1. The LLSAN was on the medial third of the RF.
2. The LLS was on the alare to the midpoint (medial half) of the RF.
3. The Zmi was on the middle third of the RF.
4. The Zmj was on the lateral third of the RF.

**Fig 5:**
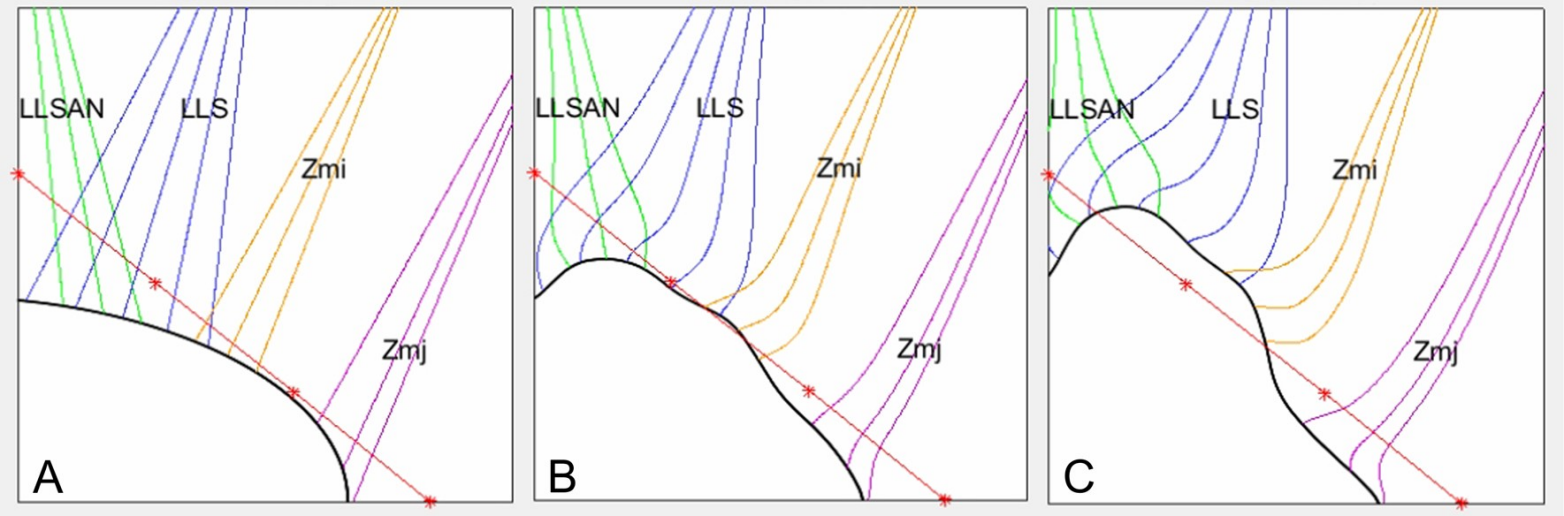
Mathematical simulation of contractions of the facial muscles along the RF (red). (A) Early stage of the simulation. (B) As the facial muscles contract, partially overlapping fibers of the LLSAN (green), LLS (blue), and Zmi (orange) began to more pull the lateral margin of the OOr (black) with greater forces according to their directions, heights, and relative proportions along the RF. (C) Movements of the muscles were more dynamic on the medial half than the lateral half of the RF, suggesting the formation and deepening of the medial half of the NLF. The RF was set to imitate the NLF from the superior point of the alar facial crease to the lateral point of the OOr at the level of the corner of the mouth. The asterisks divide the RF into its medial, middle, and lateral parts. The Zmj is indicated in purple. A simulation of the contractions of the facial muscles along the RF is presented in an online video (S4 Video).

Contractions of the facial muscles along the RF were simulated using a computational mathematical model (Fig 5). As the facial muscles contracted, the facial muscles began to pull the lateral margin of the OOr according to their directions, heights, and relative proportions along the RF. Partially overlapping fibers of the LLSAN, LLS, and Zmi contributed more to pulling the lateral margin of the OOr with greater forces. Movements of the muscles were more dynamic on the medial half than the lateral half of the RF, suggesting that hypercontractions of these facial muscles can both enhance and deepen the medial half of the NLF.

Differences between males and females regarding the prevalence of different types were analyzed. A significant difference between males and females was found in type I, which was the most common pattern in this study. Females predominated, constituting 75% of the type I specimens (12 of 16), while the numbers of females and males were similar in the other types.

The primary facial muscles responsible for producing the NLF have exhibited different height patterns in different studies and for different races (Table 1). Based on dissections, Pessa and Brown [17] reported the LLSAN to be the primary facial muscle responsible for producing the medial NLF, the LLS for defining the middle NLF, and the Zmj for accentuating the lateral NLF minimally. Those authors also considered that the LLSAN and LLS may significantly contribute to the etiology of the prominent NLF that occurs with aging. Also based on dissections, Zufferey [7] described that the medial segments are moved by direct labial tractors such as the LLSAN, LLS, and the Zmi, while the lateral segments are moved by indirect labial tractors such as the Zmj. In another dissection-based study, Snider et al. [22] found that the LLSAN inserted into the medial NLF while the LLS inserted into the middle third of the NLF. Kwon et al. [18] reported that the OOr and LLSAN were attached to the dermis of the medial NLF as a muscle complex and that the OOr, LLSAN, LLS, LAO, and the alar part of the nasalis muscle passed beneath the medial NLF in micro-CT images. They also mentioned that the Zmi and OOr were tightly attached to the dermis of the middle NLF, and that the Zmj was not attached to the dermis of the lateral NLF in micro-CT images. Thus, the present study and Kwon et al. [18] have found that the LLS passes beneath the medial third or medial half of the NLF, whereas other studies have reported that this muscle passes beneath the middle third of the NLF. Moreover, the LLS was located higher beneath the NLF in Koreans than in Caucasians.

**Table 1:** Primary facial muscles responsible for producing the NLF.

Reference	Facial muscles	Observations	Methods	Race
Pessa and Brown [17]	Medial NLF: LLSAN	Facial muscles inserting into the NLF	Dissection	Caucasian
	Middle NLF: LLS			
	Lateral NLF: Zmj minimally			
Zufferey [7]	Medial segments: direct labial tractors (LLSAN, LLS, and Zmi)	Facial muscles inserting into the NLF	Dissection	Caucasian
	Lateral segments: indirect labial tractors (Zmj)			
Snider et al. [22]	Medial NLF: LLSAN	Facial muscles inserting into the NLF	Dissection	Caucasian
	Middle third: LLS			
Kwon et al. [18]	Medial NLF: OOr and LLSAN (beneath the medial NLF: OOr, LLSAN, LLS, LAO, and alar part of the nasalis)	Facial muscles inserting into the NLF	Micro-CT images	Korean
	Middle NLF: Zmi and OOr			
	Lateral NLF: no Zmj			
This study (2020)	Medial third of the NLF: LLSAN	Facial muscles inserting into the NLF and passing beneath the NLF	Dissection and micro-CT images	Korean
	Medial third or medial half of the NLF: LLS			
	Middle third of the NLF: Zmi			
	Lateral third of the NLF: Zmj (a few fibers inserting into the NLF, main fibers passing beneath the NLF)			

The “canine” smile is caused by a dominant LLS. This pattern occurs in 35% of the population, and it is a potential candidate for BoNT-A injections. Since injecting into this muscle results in the central upper lip dropping when smiling, this intervention converts the “canine” smile into the “Mona Lisa” smile [23]. Thus, BoNT-A can be injected into the LLS at a position higher in Koreans than in Caucasians. In addition, the medial third or medial half of the NLF might be deeper in Koreans than in Caucasians due to the higher location of the LLS beneath the NLF in cases with hypercontraction of the muscle.

The effect of the Zmj on the NLF has differed between studies. Pessa and Brown [17] reported that Zmj had a minimal effect on the NLF. However, Zufferey [7] described that a concave NLF—which always results in a shallower furrow—was created by pulling on the superficial bundle of the Zmj, whereas a convex NLF—with a deep furrow—was created by pulling on the insertion of the main bundle of the Zmj on the modiolus. Zufferey [7] also reported that the Zmj was well developed in cadavers with a very marked convex NLF, even when other facial muscles were not. The present study found that the Zmj was on the lateral third of the RF or the upper part of the lateral half of the RF in most specimens, indicating that contraction of that muscle can deepen the lateral third of the NLF or the upper part of the lateral half of the NLF. In addition, a bifid Zmj was observed in 29.5% of the specimens in the present study, while its prevalence has reportedly ranged from 19.2% to 40% in previous studies [19,24,25]. Thus, it seems that the Zmj passing beneath the NLF at several heights can be one of factors resulting in various shapes of the lateral part of the NLF.

Standring [26] described that some superficial fibers of the LAO curve anteriorly and attach to the dermal floor of the lower part of the NLF. Pessa and Brown [17] reported that traction on the LAO had no effect on the NLF. However, the Zmj and LAO were located together along the RF and beneath the NLF in the present study. Shim et al. [27] described that the interlacing patterns of the Zmj with the LAO. Thus, the spatial relationship of the Zmj with the LAO should be considered when correcting the NLF.

It has been described that the Zmi assists in deepening and elevating the NLF [6,26]. However, the involvement of the Zmi in the NLF has rarely been mentioned in previous studies. Pessa et al. [8] reported that the Zmi was present in 36% of their specimens. In the present study, the Zmi was on the middle third or upper part of the middle third of the RF in all specimens, indicating that this muscle can affect a specific part of the NLF. It is therefore considered that the Zmi may affect the deepening of the NLF more in Korean than in Caucasians. Hur et al. [28] reported that muscle fibers extending from the orbicularis oculi muscle (OOc) constituted the Zmi in all specimens, which can be closely related to deepening of the NLF. Therefore, the results of the present study indicate that the middle third of the NLF—which is where the Zmi was located—can be affected by contraction of the OOc.

Pogrel et al. [29] reported that the NLF appears to be connected to muscle bundles that run both across and parallel to the fold combined with fibrous septae supporting the fat pad. The more-superficial muscle ran parallel to the fold, whereas a deeper muscle ran at right angles to it. In the present study, the LLSAN ran parallel to the NLF while the LLS, Zmi, and Zmj ran across the NLF in all specimens. In addition, although the LLS was covered partially by the LLSAN and Zmi, some superficial fibers of the LLSAN, LLS, and Zmi intermingled from different directions in the medial half and adjacent to the NLF. Therefore, the medial half of the NLF can be deeper than its lateral half due to contractions of several muscles such as the LLSAN, LLS, and Zmi. Furthermore, it is thought that these intermingled muscle fibers in different directions in the NLF may form NLFs characterized by several fine lines, and irregular and various contours.

## Conclusions

The present study utilized dissections and micro-CT to reveal the general pattern and variations of heights and spatial relationships of the facial muscles passing beneath the NLF. Beneath the NLF, several muscles intermingled and blended with the lateral margin of the OOr in different directions. These findings will be useful for understanding which muscles affect specific parts of NLFs with various contours, for reducing the NLF in aesthetic treatments, and for reconstructing the NLF in cases of facial paralysis.

## Supporting information

S1 Video A video containing serial micro-CT images of the NLF in its medial half. (MP4)

S2 Video A video containing serial micro-CT images of the NLF in its middle third. (MP4)

S3 Video A video containing serial micro-CT images of the NLF in its lateral third. (MP4)

S4 Video Heights and spatial relationships of the facial muscles acting on the NLF from the skin to the deeper area as revealed by microcomputed tomography. (GIF)
