# Supplementary figures and images for: Heights and spatial relationships of the facial muscles acting on the nasolabial fold by dissection and three-dimensional microcomputed tomography
Source: PLoS One. 2020 Aug 4;15(8):e0237043. doi: 10.1371/journal.pone.0237043 (PMC7402499; doi:10.1371/journal.pone.0237043)

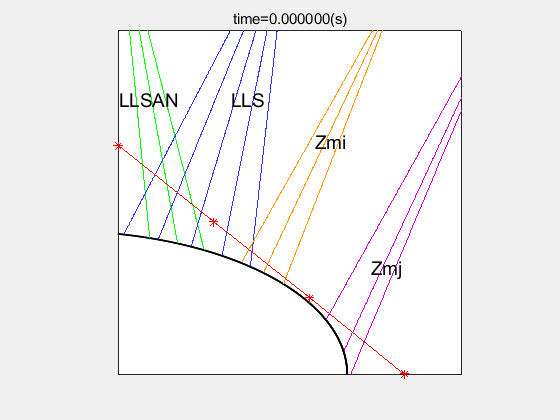

Supplement: S4 Video — (GIF) [file pone.0237043.s004.gif]
